# Determinants of Direct Costs of HIV-1 Outpatient Care in Israel

**DOI:** 10.3390/ijerph192114542

**Published:** 2022-11-05

**Authors:** Tom Rom, Itzchak Levy, Saritte Perlman, Tomer Ziv-Baran, Orna Mor

**Affiliations:** 1 School of Public Health, Sackler Faculty of Medicine, Tel-Aviv University, Tel Aviv 69978, Israel; 2 Infectious Disease Unit, Sheba Medical Center, Ramat Gan 52621, Israel; 3 Sackler School of Medicine, Sackler Faculty of Medicine, Tel-Aviv University, Tel Aviv 61390, Israel; 4 National HIV-1 and Viral Hepatitis Reference Laboratory, Ministry of Health, Chaim Sheba Medical Center, Ramat Gan 52621, Israel

**Keywords:** HIV-1, cost-prediction, Israel, economic burden

## Abstract

HIV-1 patients place an economic burden on the health system. The objectives of this study were to estimate the direct HIV-1 costs and cost-related factors of HIV-1 patients in Israel and identify cost predictors. We conducted a retrospective study of randomly selected HIV-1 patients aged ≥18 who visited a large outpatient clinic in 2015 and/or 2019. Yearly costs of physician and nurse visits, antiretroviral therapy (ART) and laboratory tests were calculated in USD using the 2020 purchasing power parities. Associations between disease characteristics and costs were analyzed using univariate and multivariable analysis. The median (IQR) total direct costs per patient per year were USD 12,387 (9813–14,124) and USD 12,835 (11,651–13,970) in 2015 (*n* = 284) and 2019 (*n* = 290), respectively. ART accounted for approximately 77% of all direct costs, followed by laboratory tests (20%) and medical visits (3%) in both studied years. Being female (USD +710), first yearly viral load <50 c/mL (+$1984) and ≥20 years with HIV-1 (USD +1056) were independently associated with higher costs. In conclusion, HIV-1 cost was stable in the studied period. Viral load and time since diagnosis were the major determinants associated with HIV-1 costs. ART and laboratory tests accounted for 97% of the costs. Therefore, these factors should be considered when planning future expenditures.

## 1. Introduction

It is globally accepted that all HIV-1 infected individuals should be treated with antiretroviral therapies (ARTs) and monitored continuously in order to reduce the risk of HIV-1 transmission and disease progression [1]. Over the past decade, a class of integrase strand transfer inhibitors (INSTIs) was added to the preferred ARTs, which initially consisted of a combination of two nucleoside/nucleotide reverse transcriptase inhibitors (NRTIs) and either a nonnucleoside reverse transcriptase inhibitor (NNRTI) or protease inhibitor (PI) [2].

HIV-1 remains an expensive illness to treat [1]. Levels of HIV-1 viral load, CD4 and mutations resistant to ARTs remain the main virological variables under constant monitoring. Other HIV-1-related variables such as time from diagnosis, adherence to treatment, coinfections, as well as age, sex, social standard and risk groups for infection could all potentially impact the cost of treatment [3,4,5,6,7].

Previous studies have demonstrated that the medical costs of HIV-1 patients are higher than controls. In several European countries and in the United States, the major component of the cost was found to be antiretroviral medications followed by inpatient care, outpatient care and laboratory costs [4,8,9]. In 2010, HIV-1 healthcare expenditure stood at 0.49% of overall medical-care spending in Italy, 0.52% in France and 0.9% in the United Kingdom [9]. Factors associated with HIV-1 costs were CD4 levels in Italy [10]; meanwhile, in England, the cost was found to be related to time since diagnosis and average costs in the first 20 years since diagnosis were lower than the costs in later years [3]. In the latter study, costs were also found to be related to HIV-1 risk groups, whereby sexual transmission was linked to higher costs compared to other risk groups [3].

In 1995, the Israeli National Health Insurance law was issued by which all eligible residents are automatically provided with medical insurance coverage. Insurance encompasses the entire range of medical services [11]. As such, all HIV-1-related costs for Israeli citizens insured by one of the four major Israeli health maintenance organizations (HMOs), including laboratory tests, ARTs and nurse and physician appointments, are covered by the government and provided to patients free of charge [12].

The planned expenditure in Israel for HIV-1 is estimated to be 0.5% of the annual health budget [13]. No recent studies estimating the direct costs related to HIV-1 in countries with social health insurance models (Bismarck model) have been published [14]. The objectives of the present study were to estimate the total annual direct costs of people living with HIV (PLHIV) followed in a large outpatient clinic in Israel, a country with a social health insurance system, and provide a spending breakdown of outpatient nurse and physician visits, laboratory tests and ARTs. Furthermore, we aimed to identify predictors associated with direct costs aiming to improve the health services provided for PLHIV. We hypothesized that increased age, longer time since diagnosis and history of multiple ART regimens would be associated with higher annual direct costs.

## 2. Methods

### 2.1. Study Design and Population

A retrospective study of randomly selected HIV-1 patients aged ≥18 visiting a large hospital outpatient clinic in 2015 and/or 2019 was performed. All patients were followed-up in a dedicated infectious disease unit located at the Sheba Medical Center (SMC) in Tel-Hashomer, Israel. SMC is a university-affiliated 1500-bed tertiary medical center. In order to study the period after the introduction of integrase inhibitors and before the COVID-19 pandemic, as well as to evaluate the effect of time, the study years of 2015 and 2019 were chosen.

Patients who had at least one nurse or physician visit and performed at least one viral load and CD4 test during the study period were eligible for this study. Patients who were not insured by one of the four HMOs, those who did not visit the clinic in one of the studied years or those with missing values in one of the three main variables (HIV-1 viral load, CD4 counts and nurse or physician visits) were excluded. The sample size was calculated using a significance level of 5% and power of 80%. In order to identify small to medium association (effect size f = 0.17) between cost and categorical variables consisting of five categories, 420 patients were required overall. Initially, 600 patients (300 in each studied year) were randomly selected.

### 2.2. Data Collection and Study Variables

Demographics, ART regimen-prescribed, blood tests and outpatient clinic visits data were extracted from the electronic medical records of SMC. Demographic data included sex, age at diagnosis, current age, birthplace, risk group of HIV-1 transmission (men who have sex with men [MSM], heterosexual contacts [Hetero], persons who inject drugs [PWID], high risk area [HRA] and unknown) and HMO. Birthplace was categorized as Sub-Saharan Africa (SSA), Eastern Europe and Central Asia (EEU/CA), Western and Central Europe and North America (WCEU/NA), Israel and other. Time since diagnosis was analyzed both as a continuous variable and utilizing a cut-off of 20 years since diagnosis based on a 2019 study conducted in England that found higher costs for patients ≥20 years post-diagnosis [3]. ART regimen-prescribed was categorized into protease inhibitors (PIs), nucleotide reverse transcriptase inhibitors (NRTIs), nonnucleoside reverse transcriptase inhibitors (NNRTIs) and integrase inhibitors (INIs). Blood tests included any drug resistance mutations identified as previously described [15], CD4 cells/mm^3^ at diagnosis, first HIV-1 viral load in copies/mL and first CD4 counts in each of the studied years. HIV-1 viral load was determined using the GeneXpert HIV-1 viral load assay [16]. The yearly number of laboratory tests (lipid and biochemistry profiles, blood count, viral load, CD4 and HIV-1 resistance) was recorded. The yearly number of nurse and infectious disease specialist visits was also extracted from the administrative database. Non-medical services, such as transportation, case management, social work services and health education, were not included. Outpatient medical care for non-HIV related conditions (i.e., comorbidities, psychopathology or substance abuse) were also not included.

### 2.3. Direct Cost Estimation

In Israel, all HIV-1 patients are exclusively followed-up in specialized units within public hospitals. Therefore, most of the costs directly associated with the management of HIV-1 can be evaluated from the electronic medical and administrative databases.

Total direct costs per patient per year were calculated by summing the yearly costs of outpatient infectious disease specialist visits, nurse visits, laboratory tests and costs of medications.

All laboratory tests were calculated as cost per test per patient. Medication costs included the cost of ARTs only. If treatment was initiated or switched during the studied year, it was recorded, and the partial yearly period for each drug was calculated. Other medications that HIV-infected patients may have been prescribed were excluded. Cost calculations were based on national price lists published in January 2020 by the Ministry of Health [17]. National price lists include all health services (i.e., medications, tests, treatments, procedures, health professional visits, etc.) and represent the official maximal prices drug companies and service providers are permitted to charge HMOs. The official national price lists are identical for all HMOs, drug companies and service providers. Costs were calculated in Israeli Shekels (ILS) and converted to US dollars (USD) using the 2020 purchasing power parities (ILS 1 = USD 3.851) published by the OECD [18]. Purchasing power parities (PPPs) are the rates of currency conversion that try to equalize the purchasing power of different currencies by eliminating the differences in price levels between countries. The basket of goods and services priced is a sample of all those that are part of final expenditures, final consumption of households and government, fixed capital formation and net exports. This indicator is measured in terms of national currency per US dollar [19].

### 2.4. Statistical Analysis

Categorical variables were summarized as frequency and percentage, and continuous variables were evaluated using histograms and Q-Q plots. Since some variables were skewed, all continuous variables were reported as the median and interquartile range (IQR) for reader ease. The Chi-square test and Fisher’s exact test were used to compare categorical variables between the two study years, while independent samples *t*-test and Mann–Whitney tests were used to compare continuous variables.

Univariate analysis was performed to study the association between direct costs and demographic, clinical and virological factors using analysis of variance (ANOVA), independence sample *t*-test and Spearman correlation coefficient for categorical and continuous variables, respectively. Factors found to be significantly associated with costs (*p* < 0.1) were included in the multivariable linear regression. The regression was evaluated to meet the assumptions. The Chi-square automatic interaction detector (CHAID analysis) was applied to identify the cost in a subgroup of patients [20].

### 2.5. Ethical Approval

The institutional review board of the Sheba Medical Center approved the study (ethical approval: SMC-7354-20). Data were obtained by chart review, and informed consent was waived owing to the retrospective nature of the study and the use of deidentified data.

## 3. Results

### 3.1. Patient Characteristics

In total, 284 patients in 2015 and 290 in 2019 were included in the study. Patient characteristics are presented in Table 1. An increase in the proportion of people infected through heterosexual contacts and a reduction in men having sex with men (MSM) infection was observed in 2019 compared to 2015. The median CD4 at diagnosis was >350 cells/mm^3^. Treatment regimens included fewer PIs and more INIs in 2019 compared to 2015. The majority of patients in both studied years were well treated, with a median viral load below 50 c/mL and median CD4 cell counts above 600 cells/mm^3^. Moreover, these CD4 counts were higher compared to CD4 levels at presentation. The overall number of patients with any resistance mutation was very low, and although statistically significant differences were found between 2015 and 2019, interpretation of these results was limited. Although the median age of the patients did not significantly differ between the studied years, the time since diagnosis was longer in those assessed in 2019.

### 3.2. Direct HIV Care Cost

Table 2 summarizes the HIV-1 cost of physician visits, nurse visits, laboratory tests and ARTs, per patient, in 2015 and 2019. ART costs accounted for approximately 77% of all direct costs in both years. Laboratory tests accounted for 20%, physician visits for 1.5% and nurse visits accounted for 0.5% of the costs.

While the median costs of nurse and physician visits were identical in 2015 and 2019, a significant increase in the costs of laboratory tests and a trend of increase in ART costs in 2019 was evident. Overall, the median cost per patient was USD 12,387 and USD 12,835 in 2015 and 2019, respectively.

### 3.3. Associations between Patient Characteristics and HIV Care Costs

To assess the associations between patient or disease characteristics and HIV costs, data from both 2015 and 2019 were combined (Table 3). Direct costs for women were found to be higher than for men (*p* = 0.036). The direct costs for patients with longer exposure to HIV-1 (those assessed ≥20 years following diagnosis) were significantly higher (*p* = 0.002) than the cost of those diagnosed <20 years ago (median of USD 12,661 compared to USD 13,086, respectively). A weak negative correlation (−0.097) was found between costs and viral load. When patients with HIV-1 viral loads below 50 c/mL were compared to those with higher viral loads, a significant negative association was observed (median cost of USD 12,820 and USD 11,236, respectively). The impact of resistance mutations on direct HIV-1 costs was difficult to estimate as the overall number of patients with drug resistance was very low.

**Figure 1:**
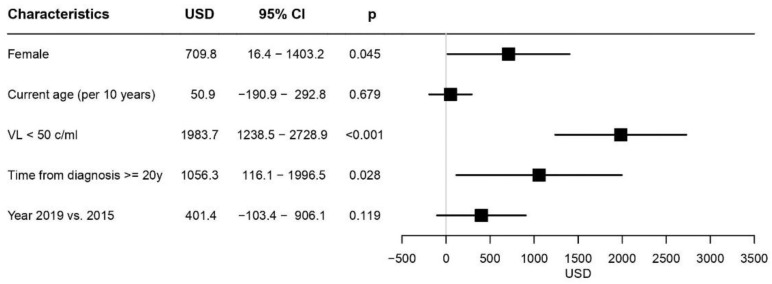
Forest plot demonstrating the factors associated with HIV-1-related direct costs as revealed by multivariable analysis. Mean additional costs, 95% CI and the significant levels associated with the cost for each factor are presented. The black boxes present the mean additional costs; the horizontal black lines present the 95% CI; the vertical grey line presents no change in costs.

Multivariable analysis (Figure 1) that included gender, age at indicated year, time from diagnosis (<20 or ≥20 years) and first viral load at indicated year (<50 or ≥50 c/mL) revealed that being female (USD +710), first yearly viral load <50 c/mL (USD +1984) and ≥20 years with HIV-1 (USD +1056) were significantly associated with higher costs.

### 3.4. In-Depth Analysis of the Factors Associated with Direct HIV-1 Cost

**Figure 2:**
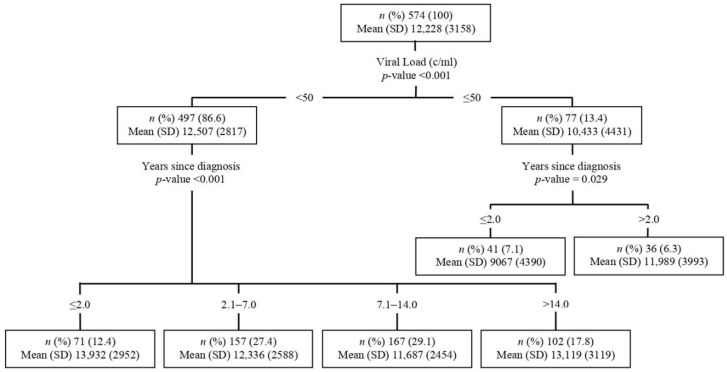
Chi-square automatic interaction detector (CHAID) analysis of factors associated with HIV-1-related direct costs. The CHAID algorithm builds a decision tree based on the full dataset by repeatedly partitioning each subset into at least two child nodes. This algorithm tries to explain the configuration of the association between potential explanatory variables and the outcome variable.

CHAID analysis was used to identify the association between a subgroup of patients and direct HIV-1 costs (Figure 2). Being well treated (viral load <50 c/mL) was found to be the most important factor associated with higher direct cost (mean USD 12,507 versus USD 10,433, *p* < 0.001). In those with viral load ≥50 c/mL, being >2 years since diagnosis was significantly associated with a higher cost (mean of USD 11,989 versus USD 9067, *p* = 0.029). No other factors were found to be significantly associated with cost in this subgroup of patients. In those who were well treated, time since HIV-1 diagnosis was also found to be the most significant factor associated with cost using 2-, 7- and 14 years as threshold values (*p* < 0.001). In those cases, being <2 years or >14 years post-diagnosis was significantly associated with higher costs (USD 13,932 and USD 13,119, respectively). Compared to 2015, 2019 was associated with higher costs in those who were ≥2 and ≤14 years post-diagnosis.

## 4. Discussion

Estimation of the economic burden of diseases is the basis for planning health expenditure. Although the prevalence of PLHIV is low [21], the direct health expenditure per patient is relatively high, leading to an economic burden on the health budget. Estimation of the real-life direct costs of HIV-1 health care and assessment of the factors associated with these costs may improve future planning of the insurer expenditure for this patient population. Based on data collected from a randomly selected cohort of patients attending a large national clinic, we estimated that the median HIV-1 cost per patient (nurse and physician visits, ARTs and laboratory tests) in 2015 and 2019 was USD 12,387 (IQR 9813–14,124) and USD 12,835 (IQR 11,651–13,970), respectively.

The proportion of the costs of nurse and physician visits remained similar in both studied years and formed only a small fraction (<3%) of all direct costs. Laboratory tests accounted for 20% of all costs, and although these costs were statistically different in 2019 compared to 2015, the difference (1%) was negligible. The laboratory costs component may play a different role in overall costs in lower-income countries that use uniform simplified treatment protocols and decentralized service distribution rather than the specialist physician management and advanced laboratory monitoring available in Western countries [22]. ARTs were the major cost component by far, accounting for 77% of the direct costs in both studied years. Previous analysis in the US also showed that three-quarters of the cost-of-care for HIV-infected patients was for ART [23]. The stability in the proportion of the direct cost components in 2015 and 2019 and the similarity in the overall expenditure across 5 years suggest that the cost values identified in this study could be applied to future cost planning unless new health technologies emerge and change the rules of the game.

The direct cost of HIV-1 was similar for people in different risk groups, so while an increase in the proportion of patients infected through heterosexual intercourse was evident in this cohort and the HIV-1 population in Israel in 2016–2018 [24], mode of HIV-1 transmission did not influence current costs. This conclusion may be different when lifetime HIV-1-related costs are assessed [25,26]. For example, in England, the estimated future costs of PLHIV were much lower when comparing MSM and heterosexuals after accounting for their different mean ages at diagnosis and survival probability [3]. Since Israel’s national health insurance law covers all residents, and co-payment is not required from any HIV-1 patient, belonging to a specific risk group, such as PLHIV (which may be associated with low socioeconomic status), should not affect direct costs, as shown in this study [27].

Gender had only a slight influence on overall costs. Yearly costs for women were shown to cost an average of USD 709.20 (CI 95%, 16.4–1, 403.2) more than men. This difference could be attributed to a longer period since diagnosis (median of 10 years versus 7.5 years for men, *p* = 0.005) and lower CD4 counts at diagnosis (median of 199 cell/mm^3^ versus 425 cell/mm^3^ in men, *p* < 0.001), which probably led to higher overall costs and ART cost (median of USD 9789 for women versus USD 9592 for men). Similarly, in Italy, it was reported that the cost for women was higher since women received a regimen containing the more expensive integrase inhibitors instead of protease inhibitors which were prescribed more frequently to men [28]. It is also possible that other gender-related biological, physiological and psychological factors may have influenced costs differently for women versus men with HIV-1 [29].

When disease-related factors were examined, it was evident that being well treated, with a viral load below 50 c/mL (in the first measurement in each studied year), was significantly associated with higher costs. These results suggest that the direct costs of a patient who is adherent to treatment are higher than the cost of a non-compliant patient. Although in Israel the cost of ART is likely not associated with patients’ compliance with treatment due to the national health insurance, the cost of ART may play an important factor in countries with different health systems. In such countries, future reduction of HIV-1 costs could be anticipated for compliant patients upon the introduction of generics [23]. Being non-adherent may also increase other costs. Due to the nature of this study population which included patients that visited the HIV outpatient clinic at least once a year, non-compliant patients were likely not included.

Another conclusion from our study is that for patients with a viral load below 50 c/mL, the main factor that affects cost is the time since diagnosis. In well-treated patients, higher costs were associated with a short period (less than two years) or long period (over 14 years) post-HIV-1 diagnosis (USD 13,932 and USD 13,119, respectively) compared to those who are in midterm (2–7 years: USD 12,336; 7–14 years: USD 11,687). Higher costs during the first 2 years post-diagnosis were already reported. In the US, it was found that 20% of patients switch therapies during the first two years after diagnosis resulting in an increase in direct medical costs [23]. On the other hand, it was demonstrated that patients with delayed ART initiation accumulate more total healthcare costs compared with patients who initiated ART early after diagnosis, highlighting the long-term benefit of rapid ART initiation [30]. Our finding that HIV-1 care costs also increase in PLHIV >14 years post-diagnosis may also reflect treatment switches and use of more expensive drug classes. It has also been shown that patients with HIV-1 who initiated PI- or NNRTI-based regimens and switched ARTs for reasons other than virologic failure used more healthcare resources and incurred greater costs relative to patients in the non-switch cohort [31].

Interestingly, the median CD4 of the patients at presentation was 400 cells/mm^3^ (IQR 211–600), suggesting that most of the patients were not late presenters characterized by CD4 counts <350 cells/mm^3^ [32]. Patients diagnosed with advanced HIV-1 disease often require more health-related resources. Therefore, late diagnosis, which characterizes a cohort of patients in Israel [24,33], could be thought to increase HIV-1 costs. However, in studies that included a large proportion of ART recipients with excellent virological control, CD4 cell count at presentation was no longer a major factor for higher costs [34].

The majority of patients in this randomly selected cohort were Israeli-born individuals. More men (>80%), mainly MSM (>60%), were included; however, a slight decrease in the proportion of MSM and an increase in those belonging to the heterosexual transmission group in 2019 was noted. This pattern is similar to the reported risk group proportions and changes in the prevalence of the various risk groups of newly diagnosed individuals in those years [24]. In addition, variation between patients treated at different large outpatient clinics is not expected due to the social health insurance system in Israel. Therefore, the studied cohort is likely representative of the treated HIV-1 patients across Israel in 2015 and 2019. Generally, the patients in 2019 were slightly older and were HIV-1 positive for a longer time, suggesting the aging of the PLHIV population. With the use of ART, an increase in life expectancy is expected, as already reported by others [35].

This study has several limitations. First, not all costs related to HIV-1 patients were calculated. For example, hospitalization or services provided outside of the clinic, as well as costs of social or psychological services, were not included. Second, due to the 4-year time interval between evaluations, we preferred to consider the few patients that were included in both study years as individual cases. Third, the actual ART costs negotiated between each HMO and the respective drug companies and service providers are unknown. The costs presented are the official maximal costs that can be requested and are updated annually by the Ministry of Health. Using this official national price list enabled the standardization of the costs for comparison purposes and the identification of cost-related factors. Fourth, costs were calculated based solely on data included in the medical records available in the AIDS unit. This may be different from what was actually consumed by each of the patients. However, due to the provision of HIV-1 health-related services that are fully covered by national health insurance and the selection of patients who attended the clinic in the study years, it can be assumed that the overall cost presented is not an overestimation.

## 5. Conclusions

To the best of our knowledge, this is the first study in Israel that assessed direct HIV-1 costs. Our findings show that the costs of physician and nurse visits are marginal compared to the costs of ART and laboratory tests and thus do not support the need to decentralize HIV-1 treatment centers in hospitals in order to reduce direct HIV-1 costs. In the future, when new therapies are included in the health basket provided by the insurer, the established cost of both innovative therapies and generic drugs should be re-estimated. The introduction of generic and innovative drugs will likely mutually impact the overall ART cost; thus, the overall direct health care cost for HIV-1 patients will be similar.

## Figures and Tables

**Table 1:** Characterization of the patient population in 2015 and 2019.

	Year		*p*-Value
	2015	2019	
	(*n* = 284)	(*n* = 290)	
Gender, *n* (%)			0.298
Male	244 (85.9)	240 (82.8)	
Female	40 (14.1)	50 (17.2)	
Age at Diagnosis (year), Median (IQR)	34 (27–41)	34 (27–41)	0.658
Age at Indicated Year (year), Median (IQR)	42 (35–51)	44 (36–53)	0.048
Time from diagnosis (year), Median (IQR)	7 (3–12)	9 (4–14)	0.040
Place of Birth, *n* (%)			0.116
Israel	192 (67.6)	181 (62.4)	
EEU/CA	42 (14.8)	56 (19.3)	
SSA	19 (6.7)	31 (10.7)	
WCEU/NA	12 (4.2)	6 (2.1)	
Other	19 (6.7)	16 (5.5)	
Risk Group, *n* (%)			0.007
MSM	197 (69.4)	162 (55.9)	
Hetero	35 (12.3)	59 (20.3)	
PWID	28 (9.9)	29 (10)	
HRA	22 (7.7)	35 (12.1)	
Unknown	2 (0.7)	5 (1.7)	
HMO, *n* (%)			0.935
Clalit	138 (48.6)	135 (46.6)	
Maccabi	121 (42.6)	131 (45.2)	
Leumit	14 (4.9)	13 (4.5)	
Meuhedet	11 (3.9)	10 (3.4)	
ARTs, *n* (%)			
PIs	66 (23.2)	7 (2.4)	<0.001
NRTIs	273 (96.1)	287 (99.0)	0.053
NNRTIs	60 (21.1)	44 (15.2)	0.081
INIs	170 (59.9)	269 (92.8)	<0.001
Resistance, *n* (%)	11 (3.9)	2 (0.7)	0.010
CD4 at Diagnosis (cells/mm^3^), Median (IQR)	421 (248–646)	386 (173–572)	0.158
First CD4 (cells/mm^3^), Median (IQR)	667 (511–849)	671 (468–866)	0.820
First VL <50 c/mL, *n* (%)	241 (84.9)	256 (88.3)	0.230

IQR—interquartile range; SSA—Sub-Saharan Africa; EEU/CA—East Europe and Central Asia; MSM—Men who have sex with men; Hetero—Heterosexual contacts; PWID—persons who inject drugs; HRA—High-risk area; HMO—Health maintenance organization; PI—Protease inhibitors; NRTIs—Nucleotide reverse transcriptase inhibitors; NNRTIs—Nonnucleoside reverse transcriptase inhibitors; INI—Integrase inhibitors; First CD4—CD4 value at the first measurement taken at year; First VL—Viral load value at the first measurement taken at year.

**Table 2:** Median (IQR) costs ($) per HIV patient per year.

Year	Physician Visits	Nurse Visits	ART	Laboratory Tests	Total Cost
2015	197 (119–237)	46 (46–69)	9571 (7562–11,700)	2093 (1883–3144)	12,387 (9813–14,124)
2019	197 (119–237)	46 (46–69)	9592 (9427–10,980)	2120 (2093–3158)	12,835 (11,651–13,970)
*p*-value	0.817	0.993	0.089	0.040	0.040

**Table 3:** Univariate analysis of disease-associated parameters and direct HIV-1 cost.

	Total Cost ($) per Patient/Correlation Coefficient	*p*-Value
Gender, Median (IQR)		
Male (*n* = 484)	12,484 (10,489–14,027)	0.030
Female (*n* = 90)	12,938 (11,849–14,038)	
Age at Diagnosis (year), r_s_	0.033	0.424
Age at Indicated Year (year), r_s_	0.07	0.093
Time from diagnosis (year), r_s_	0.032	0.448
Less than 20 years, Median (IQR)	12,661 (10,667–13,833)	0.002
20 years and more, Median (IQR)	13,086 (11,823–15,752)	
Place of Birth, Median (IQR)		0.482
Israel	12,716 (10,707–14,025)	
SSA	12,939 (10,626–14,728)	
EEU/CA	12,539 (10,707–14,008)	
Other	12,449 (9788–14,017)	
Risk Group, Median (IQR)		0.435
MSM	12,449 (10,229–14,025)	
HETERO	12,835 (10,707–14,017)	
PWID	12,948 (11,172–14,087)	
HRA	12,929 (10,707–14,462)	
Unknown	12,296 (10,910–13,784)	
HMO, Median (IQR)		0.986
Clalit	12,554 (10,112–14,084)	
Maccabi	12,818 (10,883–13,991)	
Meuhedet/Leumit	12,912 (9962–14,551)	
Resistance, Median (IQR)		0.428
No	12,815 (10,707–14,027)	
Yes	9312 (7758–14,331)	
CD4 at Diagnosis, r_s_ (*n* = 358)	−0.047	0.375
First CD4, r_s_	−0.057	0.174
First VL, r_s_	−0.097	0.020
Less than 50 copies/mL, Median (IQR)	12,820 (10,850–14,024)	<0.001
50 copies/mL or more, Median (IQR)	11,236 (7498–14,049)	

IQR—interquartile range; r_s_—Spearman’s correlation; SSA—Sub-Saharan Africa; EEU/CA—East Europe and Central Asia; MSM—Men who have sex with men; HETERO—Heterosexual contacts; PWID—persons who inject drugs; HRA—High-risk area; HMO—Health maintenance organization; First CD4—CD4 value at the first measurement taken at year; First VL—Viral load value at the first measurement taken at year.

## Data Availability

Data will be made available for reasonable requests.
